# Action-Oriented Study Circles Facilitate Efforts in Nursing Homes to “Go from Feeding to Serving”: Conceptual Perspectives on Knowledge Translation and Workplace Learning

**DOI:** 10.1155/2012/627371

**Published:** 2012-09-06

**Authors:** Albert Westergren

**Affiliations:** The PRO-CARE Group, School of Health and Society, Kristianstad University, 291 88 Kristianstad, Sweden

## Abstract

*Background*. Action-oriented study circles (AOSC) have been found to improve nutrition in 24 nursing homes in Sweden. Little, however, is known about the conceptual use of knowledge (changes in staffs' knowledge and behaviours). *Methods*. Qualitative and quantitative methods, structured questionnaires for evaluating participants' (working in nursing homes) experiences from study circles (*n* = 592, 71 AOSC) and for comparisons between AOSC participants (*n* = 74) and nonparticipants (*n* = 115). Finally, a focus group interview was conducted with AOSC participants (in total *n* = 12). Statistical, conventional, and directed content analyses were used. *Results*. Participants experienced a statistically significant increase in their knowledge about eating and nutrition, when retrospectively comparing before participating and after, as well as in comparison to non-participants, and they felt that the management was engaged in and took care of ideas regarding food and mealtimes to a significantly greater extent than non-participants. The use of AOSC was successful judging from how staff members had changed their attitudes and behaviours toward feeding residents. *Conclusions*. AOSC facilitates professional development, better system performance, and, as shown in previous studies, better patient outcome. Based on a collaborative learning perspective, AOSC manages to integrate evidence, context, and facilitation in the efforts to achieve knowledge translation in a learning organisation. This study has implications also for other care settings implementing AOSC.

## 1. Background

Promoting nutritional health equity among at risk older populations in nursing homes is of importance. Effective interventions that reduce the risk of undernutrition can help to ensure that people stay healthier in old age. Findings from a couple of studies indicate inequity between the nutritional care provided to patients in hospitals and elderly people in nursing homes. Two surveys conducted in 2007 indicate that patients in hospitals and at risk of undernutrition are more likely to get oral supplements (43–54%) [1] than elderly persons at risk of undernutrition in nursing homes (14–19%) [2]. It is plausible that appropriate nutritional care for older people in nursing homes requires educational interventions.

This study is a part of a larger project that has shown, in a before–during–after controlled trial, that by implementing action oriented study circles (AOSC) for the staff in nursing homes, knowledge translation (KT) regarding eating and nutrition was achieved in terms of instrumental outcomes (had a positive impact both on provider behaviour and on patient outcomes) [2, 3]. The means by which this was achieved is believed to be inconsistent and complex, and what occurred can be described as a “black box phenomenon” [4]. It is presumed that evidence, context, and facilitation influenced the KT in the previous studies [2, 3], but this remains to be explored. This study puts action-oriented study circles (AOSC), a pedagogical method for work based collaborative learning, and KT into a theoretical context. It also looks into the “black box” of KT and reveals some of its content.

**Knowledge Translation**

It is a challenge to achieve improvements in nutritional care in an environment that is complex and evolving, and there is a tendency to measure the success of projects only in terms of instrumental outcomes of KT (better system performance (care) and measurable change in patient outcomes (health)). However, besides instrumental outcomes, also conceptual outcomes are of interest when it comes to improving nutritional care. The conceptual outcomes describe changes in understanding, knowledge and attitudes [5]. It is suggested that research translation should be used as an intermediate outcome, while patient outcomes and provider behaviour are endpoint outcomes [6, 7].

KT can be defined as “the exchange, synthesis, and ethically-sound application of knowledge—within a complex system of interactions among researchers and users—to accelerate the capture of the benefits of research … through improved health, more effective services and products, and a strengthened health care system” (Canadian Institutes of Health Research, cited in [8] page 1). KT is a processual phenomenon that is dynamic and interactive [9]. The purpose of KT is to decrease the gap between what is known from research and knowledge synthesis, and the implementation of this knowledge by key stakeholders, in order to improve care delivery, efficiencies of the health care system and health outcomes [5]. Graham and Tetroe [10] highlight the importance of improving our knowledge about KT. For instance, more research is needed that focuses on developing theory-based KT interventions and on testing their effectiveness.

**The Organisational Influence and the PARiHS Framework**

In this study, the success of the implementation of change is believed to be linked to the nursing home organisation. Thus, in order to succeed, the type of organisation and decentralised decision making, in relation to the teams, are regarded as important [11]. A framework for successful implementation that incorporates organisational factors (such as facilitation and context) is the PARiHS framework (Promoting Action on Research Implementation in Health Sciences), developed since 1998 [12, 13]. The framework can be used to theory-based KT, and to better understand the “black box” of implementation. According to Kitson and colleagues [12], a “successful implementation” (SI) of new ideas (evidence, guidelines, etc.) is a function (f) of the interrelations between three key elements—evidence (E), context (C), and facilitation (F): SI = f (E, C, and F).

The dimension *Evidence* includes research, clinical experience, patient experience, and routine data. Thus, it is important to not only consider evidence within the “evidence-based medicine” framework. Instead, in the PARiHS framework, also other sources of knowledge are considered, including research evidence, clinical experience, professional craft knowledge, patient preferences and experiences, and local information [12]. Graham et al. [5] conceptualise, in relation to KT, that “knowledge” can be empirically derived (research based) but also encompass other forms of knowing, such as experiential knowledge. Both local and external knowledge creation or research can be integrated in the knowledge to action process [5]. For instance, local research can be carried out in order to determine the magnitude of the problem and the care gap [5]. The *Context* in a care setting is made up of several factors, factors that are interdependent [12, 14]. Among others such as leadership, culture (prevailing beliefs, values of relationships, teamwork, power, and reward systems), feedback processes (information sharing), and organisational slack (human resources, space, time) are part of the context [14]. *Facilitation* includes purpose, role, skills, and attributes. The subelement “Purpose” (in the *Facilitation* dimension) can be technical or holistic. “Technical” means introducing a discrete method, while “holistic” means sustaining and enabling personal development and system transformation [12]. Facilitation refers to the process of “enabling (making easier) the implementation of evidence into practice” and “facilitation is achieved by an individual carrying out a specific role (a facilitator), which aims to help others” ([13] page 579). The facilitator has an appointed role, internal or external to the organisation, in which the change is being implemented. Facilitators are said to have a key role in helping teams and individuals understand what and how they need to change practice. The role of facilitator is more about helping and enabling than persuading or telling [13]. In the two previously mentioned studies [2, 3], the intervention with study circles was theory based in the PARiHS framework.

The success of the implementation of change is believed to be linked to the professionals' needs and motivation and to the fact that people change their behaviour on the basis of experienced problems in practice [11]. Thus, in order to achieve improved nutritional care, a pedagogical method is needed to increase professional competence, to better serve the care recipients, to meet external expectations (including pressure from colleagues), and to inspire to change by learning through social interaction. Action-oriented study circles might have these qualities.

**Action Oriented Study Circles**

By adding “action oriented” to “study circles,” I want to emphasise that it is not only “studies” that are expected but also “action” (knowledge to act). Action-oriented study circles (AOSC) is a pedagogical method that might increase the likelihood of achieving KT, as it emphasises work-based learning in the local social context. Often, different theoretical perspectives must be considered in order to develop a good KT [11], and using AOSC makes it possible to integrate pedagogical, social, and organisational theoretical perspectives. Furthermore, the study circle methodology can be linked to the PARiHS framework in that the facilitation aspect is very much connected to enabling self directive learning and cooperative learning approaches [12]. Facilitative learning approaches that are student centred and problem based emphasise critical reflection and focus on experimental learning, and by achieving local consensus they have the potential to enhance changes in practice culture, as well as evidence-based improvements.

The “birth year” for the study circle is regarded to be 1902. It started in the temperance movement, and its instigator was Oscar “with the beard” Olsson. It has since then been the core method used by the Workers' Educational Association (ABF) in Sweden [15]. The idea of the study circle can be connected to Vygotsky's ideas about cultural mediation and internalisation, namely, that by interacting in a group, individuals come to share the knowledge of a culture. In this way, knowledge becomes internalised, one “knows how” [16]. Also Piaget's thoughts on “cooperative relations” are applicable to the ideas behind study circles. In cooperative relations, power is evenly distributed between participants so that a symmetrical relationship emerges. In cooperative relations, authentic forms of intellectual exchange become possible; each partner has the freedom to project his or her own thoughts, consider the positions of others, and defend his or her own point of view. According to Piaget, the knowledge that emerges is open, flexible, and regulated by the logic of argument rather than determined by an external authority [17]. Study circles also have similarities with problem-based learning (PBL), and pedagogical research has shown that learning is enhanced when the student is active [18, 19].

The fundamental concepts of the study circle are voluntary, informal, and participant-centred learning in which flexibility of format and structure is important [20]. If pedagogical models are divided into the persuasion model, the information model and the discussion model, the study circle belongs to the discussion model. The discussion model means that the participants engage in conversations, identify and analyse problems, and take actions [21]. In addition, the study circle places great emphasis on the broader environmental and social context in which individuals work and where the interventions for change are expected to occur. By using a discussion model for learning, one assumes that knowledge acquisition is a collaborative process and that individual understanding is rooted in social interaction. Because of its simple, flexible structure, and its capacity to address contextual factors, the study circle could serve as a model for educational interventions in care and service. The overall aim of study circles is to identify key problems and to learn how to master them [22]. Several positive experiences have been described by participants in study circle activities arranged within the popular education framework in Sweden (folkbildning), such as personal development, increased self-esteem, changed ways of thinking, increased courage, learning how to study, increased social and cultural competence, and improved cooperation and solidarity [15].

Study circles are becoming increasingly popular in Sweden in care and service. The Swedish Institute for Health Sciences (Vårdalinstitutet) [23], for instance, has several manuals for study circles for different areas such as dementia, ethics, culture, eating and nutrition, palliative care, pain, stroke, and elderly health. However, study circle interventions in care and service have been explored only in a few studies [2, 3, 22, 24–28], and some of these have focused on nutrition in nursing homes and instrumental outcomes of KT [2, 3, 28]. It was shown that study circle interventions with focus on nutrition in nursing homes can improve the care (more residents with risk of undernutrition get the right treatment) [2, 3] and the care recipients' outcome (decreased number of residents with undernutrition) [2, 3, 28] in a short- [2, 28] and long-term perspective [3]. Wallin [7] states that “if we want to understand what strategies are working in changing practice to be more evidence-based, then we must test these strategies” (page 579). Thus, research has shown that study circle interventions improve instrumental outcomes, and now we need to show what impact they have on staff knowledge and attitudes, that is, conceptual outcomes. Furthermore, Wallin [7] highlights that there is a need for examining the PARiHS framework through intervention studies. Thus, when exploring the process of KT with focus on nutrition using the AOSC methodology, it should be done in relation to a theoretical framework.

This study reports on the use of AOSC as an intervention to promote the conceptual use of new knowledge concerning eating and nutrition in nursing homes in Sweden, using the Promoting Action on Research Implementation in Health Sciences (PARiHS) framework as the explanatory theoretical model.

**Aim**

The aims were to explore nursing home staffs' views of participating in action-oriented study circles focused on eating and nutrition, to compare participants with non-participants, and to describe goals set by study circle participants.

## 2. Methods

In this study, both quantitative methods (structured questionnaires, comparisons between groups and over time) and qualitative methods (knowledge-to-action goals and focus group interviews) were used. The quantitative data focused on the content of the AOSC, the participants and non-participants self-evaluation of their knowledge about eating and nutrition, and on how they thought the management acted upon ideas regarding food and mealtimes. The qualitative methods were used to explore nursing home staffs' views of participating in AOSC and to capture the knowledge-to-action goals set by the study circle participants.

### 2.1. Description of the Project as a Whole

The project as a whole is built up by one all-embracing “knowledge-to-action process” [5] as well as by smaller action processes within each study circle. The knowledge-to-action process described in this study is based on Graham et al. [5]. First of all, the problems with nutrition were identified by a large survey of nutritional status and nutritional care in 2005, capturing the care gap [29]. Following this, a study circle manual, with reference to relevant literature, was developed [30]. Thus, based on the manual, as well as on the findings from the survey in 2005, the identified knowledge was adapted to the local context by the participants in AOSC. In addition, each study circle had the possibility to further adapt the knowledge to their particular setting. Barriers to using the knowledge were continuously assessed by regular evaluations and through dialogues with the front-line staff. Within each study circle, interventions were selected, tailored, and implemented to promote the use of knowledge (i.e., implement the change). The instrumental use of knowledge was monitored in 2007, by repeating the survey of nutritional status and nutritional care, and comparing findings with baseline data from 2005 [2]. The conceptual use of knowledge was monitored every six months (during the two years in which the study circles were going on). To evaluate whether sustained instrumental knowledge use was achieved, a final survey (with the same methodology as in 2005 and 2007) was conducted in 2009 [3].

### 2.2. This Study

The focus of this study was the conceptual use of knowledge. In order to evaluate the process of change and the important ingredients in that process, both qualitative and quantitative methods were used, that is, focus group interviews and questionnaires. Wallin [7] states that qualitative research is particularly helpful in uncovering why something happens and in identifying the active ingredients of an intervention. Further, Titler [31] advocates using “natural experiments,” in the study of KT, as it makes it possible to capture “real world” initiatives that otherwise are not feasible to investigate or would be too costly [31].

### 2.3. Sample

The study involved 24 nursing homes (with one to nine units in each nursing home) and three home care areas. The number of participants from each nursing home or home care area was in median 24 (range 6–74 participants).

This study describes the results from three to some extent overlapping samples:

- Participants (*n* = 592) in all study circles (71 study circles, about 8 participants/AOSC) continuously evaluated their experiences (structured questionnaires) (during spring 2006 till autumn 2008) (Table 1). The target group for the study circles was front-line staff working in the nursing homes (long-term care facilities) as well as staff working in the kitchens (Table 2).
- AOSC participants (*n* = 74) and AOSC non-participants (*n* = 115), staff working in nursing homes attending an education day (spring 2007) with focus on eating and nutrition. Thus, the non-participants were those that no yet had been involved in AOSC but were attending the education day.
- At the end of the project period (December 2008), a focus group interview was carried out with project leaders (*n* = 2), study circle leaders (*n* = 5), both leader and participant (*n* = 1), and participants (*n* = 4) (in total *n* = 12). The managers contacted the previous AOSC participants and asked if they would like to participate in the focus group.

### 2.4. Intervention: Action Oriented Study Circles

Inspired by Kitson and colleagues' [12] ideas about the three key elements (evidence, context and facilitation) for a successful implementation of evidence and knowledge into practice, the AOSC intervention is here described. 

#### 2.4.1. Evidence

 A manual for the study circles was developed by Elisabet Rothenberg and the author of this paper (AW) [30]. Six themes were discussed: (1) the importance of food for the care recipient, (2) difficulties with eating, (3) routines, tools and responsibility, (4) food as medicine, (5) food hygiene and (6) when the mealtime becomes a question of life and death. In the manual, references were made to chapters in two books from the National Food Administration [32, 33], as well as to scientifically based texts online written by researchers in the field. Besides this, the staff also had access to results from the survey of nutritional status and care done in 2005 [29].

The pedagogical method included the identification of specific nutritionally related problems that the participants decided to discuss and a brainstorming session about ways to solve the problem in their own context, at their own unit. A structured plan of action was developed in order to achieve the necessary changes, bridging the gap between knowledge and care. The participants were encouraged to use the power of the group to achieve the changes. More than one study circle could be held at each unit.

#### 2.4.2. Facilitation

 The facilitation that was provided can mainly be considered “holistic,” that is, sustained and enabled personal development and system transformation [12]. External facilitation was provided by the project leaders for the study circle intervention and the management. The project leaders coordinated the circles and evaluated them regularly (every six months). They also ordered and distributed the material and had discussions with politicians and managers. The project leaders were not involved in the study circles but could be invited to the groups for discussions. The intervention was supported by the management. Thus, managers ensured that the staff got time to prioritise the study circles. Internal facilitation was provided by the study circle leaders, who acted as facilitators for the participants. 

The role of the study circle leader was to administer the circles, facilitate discussions, and ensure that the participants focused on the issues. The study circle leader did not need to be an expert in the focused field. As Strombeck ([20] page 10) states, “the function of the leader is to help promote a positive environment in which participants are encouraged to analyze, discuss and critically examine what they have read.” The study circle leaders were provided training in their forthcoming role. Before the first six months of study circles the leaders were given half a day of training, and after the six-month evaluation, it was decided that the new leaders should be given a full day of training. In addition, it was decided after six months that the management should work towards recruiting homogenous groups (persons from the same nursing home and ward) instead of having the groups be heterogeneous (persons from different nursing homes and wards), and that instead of having six meetings that last for one and a half-hour each, there should be three meetings that last for three hours each. Thus, most (about 65) of the AOSC had three meetings that lasted for three hours each.

Based on the study circle manual and related references, the individuals made decisions about the value, usefulness, and appropriateness of the particular knowledge to their settings and circumstances. The topic area, the particular knowledge to implement, was agreed upon (local consensus) in each study circle and arose from the awareness that there was a need to improve the older persons' experiences from mealtimes and outcomes relating to nutrition. Each of the study circle teams was required to define the problem, work on how to create a potential for change, and then hopefully improve the older persons' experiences and outcomes. Thus, they tailored or customised the knowledge to their particular context. What knowledge to implement could vary from circle to circle, and it was not at all stressed that they should focus on screening for undernutrition and on what measures to take for those who were at risk for undernutrition, that is, the outcomes measured in the previously mentioned two studies [2, 3] of which this study is a part.

### 2.5. Data Collection

Structured questionnaires after each study circle, focusing on content, level of difficulty [23], and knowledge were used (sample a). The participants retrospectively rated their knowledge, thus, they rated their knowledge before the intervention and after in the same questionnaire. Besides this, qualitative data regarding goals for quality improvements set within each study circle (*n* = 71) were collected. 

In addition, a questionnaire was given to participants attending an education day (sample b). They filled in the questionnaire before the education began. Both staff that previously had been AOSC participants (*n* = 74) and staff that no yet had been involved in AOSC (*n* = 115) answered the questionnaire that focused on knowledge development and perception of management engagement.

Within the focus group (sample c), experiences from participating or leading study circles were discussed. The interview took three hours. Each participant gave written consent to participate and agreed to have the interview tape-recorded. The interview was transcribed verbatim and analysed using conventional and directed content analysis [34–36]. 

### 2.6. Analysis

#### 2.6.1. Quantitative analysis

 Parametric and non-parametric statistics were used depending on the level of data and based on unpaired comparisons between two groups. The following tests were applied: *T*-test, Chi-square test, Wilcoxon signed ranks test, and Mann Whitney *U* test. The level of statistical significance was set at *P*-value < 0.05. Analyses were performed using PASW Statistics 18.0.

#### 2.6.2. Qualitative analysis

 Conventional (latent and manifest) and directed content analysis was chosen as the method of analysis for the focus group interviews in order to clarify deeper or latent meanings in the text [34, 36]. Both manifest and latent content analysis include an interpretation of the text material, although both vary with regard to interpretation depth and abstraction level [35]. 

The text was read several times in order to gain an overall understanding. On the basis of the first readings, distinguished patterns emerged from the texts. These patterns made up the first results: “the naive understanding”. These first thoughts and reflections on the text contents were recorded in the margins and were used continuously during the remaining stages of the analysis. Step-by-step, the material was analysed with the aim of the study in mind. “Meaning units” were then identified, that is, assertions from the contents of the text. Meaning units with similar content were organised into different areas (categories) with the study aim in focus. The meaning units were condensed (the sentences were shortened while preserving the core) [34]. Subcategories were created for sorting out the analysed material. At the last stage, the subcategories were linked together into two main categories that in turn were linked to a core category. Afterwards, the concepts from the PARiHS framework [12] were deduced to the qualitative findings (directed content analysis) [36]. Categories at different levels were ordered in relation to *Evidence*, *Context*, *Facilitation* and *Successful Implementation*. 

### 2.7. Ethics

The ethics for conducting scientific work were followed. This study was approved in each municipality. The respondents were asked for informed consent. Both verbal and written information were given and respondents were guaranteed confidentiality (no personal identification numbers or names were collected). Within the focus group, each participant gave written consent to participate and agreed to have the interview tape recorded. As the study was part of an overall quality development project, no formal approval by an ethical committee was required, according to the Swedish Act concerning the Ethical Review of Research Involving Humans [37].

## 3. Results 

### 3.1. All Study Circles

During three years (2006–2008), 592 persons from 24 nursing homes participated in the study circles, according to the filled-in and returned questionnaires. There were 71 study circles in total with an average of eight persons per circle (Table 1). 

566 women (96%) and 26 men (4%) participated, with a mean age of 45 years (min 20, max 66, q1–q3 39–51 years). The most common profession participating in study circles was auxiliary nurses or nursing assistants (80%). Most participants had been working in their current line of work for more than 20 years (Table 2). 

The most common combination of staff in each circle was one person working in the kitchen, who usually also was the circle leader, and seven auxiliary nurses or nursing assistants. The staff members attending each study circle usually came from the same unit, except during the first six months of study circles (six study circles).

#### 3.1.1. Evidence and Knowledge

Of those attending the study circles, 98% regarded the content as interesting/very interesting, 94% thought that it was relevant/very relevant and 92% considered that the difficulty level was easy/neither difficult or easy (Table 2).

When participants retrospectively rated their knowledge about food and nutrition before and after having participated in the study circle, there was a significant improvement. Before the study circle, 72% stated that they had sufficient or great knowledge and afterwards 96% stated the same thing (Table 3).

#### 3.1.2. Action Orientation Goals for Quality Improvement

The action orientation of the study circles included the identification of specific nutritionally related problems that the participants decided to discuss and brainstorm in order to find ways to solve the problem in their own context, at their own unit. A structured plan of action was developed by the participants in order to achieve the necessary changes. Thus, each study circle worked with setting goals to work towards within each group's workplace. These goals could be grouped into environment, food, hygiene, routines, shortening overnight fast, the ability to choose, and cooperation (Table 4).

### 3.2. Comparisons between Those Participating and Those Not 

#### 3.2.1. Facilitation and Knowledge

In total 189 structured questionnaires were returned during the education day and it is unclear how many did not answer them. There were no differences in age, gender, or in number of years in current workplace between AOSC participants and non-participants. However, there was a significant difference in professions, with more cook/kitchen helpers and less registered nurses among the AOSC participants (Table 5). 

More among those who had participated than among those who had not participated in the study circles agreed totally/partly that the management was engaged in and took care of ideas regarding food and mealtimes (80% and 66%, *P*-value = 0.009). In addition, more of those who had participated in study circles than among those who had not felt that they had great or sufficient knowledge about the field (69% versus 63%, *P*-value = 0.044) (Table 5).

### 3.3. Focus Group Interview

The analysis of the focus group interview with project leaders (*n* = 2, one man and one woman), circle leaders (*n* = 5, all women of whom two worked in kitchen), and participants (*n* = 4, all women) in the study circles resulted in one core category: “nutritional knowledge translation expressed as *going from feeding to serving*.” This was the most concluding description of what was achieved. This core category captures an awareness that includes both the meaning of the mealtime environment, being together, and staff that is service minded, lays the table and presents the food in an appealing way. It also captures the two categories that it was built up from (Table 5).

- Socioculturally Mediated knowledge spread.
- Facilitation of knowledge spread and sustainability

#### 3.3.1. Socio-Cultural Mediated Knowledge Spread

The main impression from this category was that the socio-culturally mediated knowledge spread contributed to a shared vision among the staff. This category was built up from three subcategories (Table 6).

- Together and from the same place we are stronger.
- Getting together broadens one's perspective and improves the work atmosphere.
- Through discussions, the gap between knowledge and practice can be bridged.

The participants expressed both that one person achieves less change than many and that one study circle achieves less change than many. “One swallow does not make a summer” can here be used as a metaphor (developed by the researcher) to illustrate that one single person who attends a training was described as having difficulties in achieving changes in the workplace. Staff members say that attending a training day as the only representative from their workplace made it difficult to gain interest and understanding for what they had learned and what they wanted to change. It became clear that the staff experienced the study circle as the superior method, since it meant that several persons from the same setting came together and discussed and set goals that were adapted to their own workplace, their own context. In addition, many participants expressed a preference for homogenous groups rather than heterogeneous groups. Study circles with participants from different workplaces made it difficult for participants to mutually agree on the same goals, and more difficult to communicate the goals to their own workplace. Furthermore, “rings on the water” can be used as a metaphor to say that the more study circles that had taken place at the same workplace, the greater was the possibility of achieving positive changes. To summarise, “together and from the same place we are stronger.”

Broadening one's perspective signifies that the participants in the study circles experienced a greater understanding for each other and each other's work tasks, saw new dimensions of their work, and communicated more with each other. The understanding between the staff improved, especially between those working in the ward and those working in the kitchen. The work atmosphere improved. 

The participants felt that the gap between knowledge and practice could be bridged through the discussions in the study circles. The ward culture and personal engagement were thought to have improved as a consequence of the knowledge-focused discussions between colleagues. The staff felt that they had gained a deeper knowledge about food and mealtimes, knowledge that was based on evidence and the groups' experiences, and not only on the individuals' own experiences. They also said that they had changed their attitudes regarding the food, from being negative to being more positive. The food and mealtime was regarded more and more important. In addition, they described how incorrect routines or a negative ward culture could be improved through relearning. 

#### 3.3.2. Facilitation of Knowledge Spread and Sustainability

The category “facilitation of knowledge spread and sustainability” was built up from three subcategories (Table 6):

- action-oriented goals
- facilitation by anchoring and feedback
- facilitation of sustainability

It was seen as valuable to set and evaluate goals within the study circles. At wards where there had been many study circles, it became, in the end, difficult to set new goals. Participants also described different ways of making the staff more aware of the goals, for instance, to write down all goals in a document that was framed and hung in a place where everyone could see it. Staff members also talked about how they informed new staff and substitutes about the goals. Many improvements were also described as being an effect of the set goals, improvements that for instance meant a calmer and more homelike mealtime environment, and better measuring and followup of weight, and so forth. 

Facilitation by anchoring and feedback of results was seen as important. A lot of information and persuasion were required in order to make the management understand the importance of the study circles. When management got feedback of the results from each six-month evaluation, their motivation to support and back up the intervention increased. 

Facilitation of sustainability in order to maintain and achieve new improvements in practice was mainly focused on the time after finishing the project. It was clear from the focus group interviews that both the study circle participants and the leaders wanted to maintain and further develop the improvements that were initiated or achieved from the study circles. Suggestions were put forward regarding how the work could be continued, for instance, by discussing food and mealtimes at workplace meetings and to continue with study circles for those who had still not participated in any (Table 6). 

### 3.4. Directed Content Analysis

The deductive/directed content analysis, using the PARiHS framework, of the findings from the conventional/inductive analysis showed that the category “socio-culturally mediated knowledge spread” related to the components context and evidence, while the category “Facilitation of knowledge spread and sustainability” was related to the two categories facilitation and evidence. The core category “nutritional knowledge translation expressed as *going from feeding to serving*” was considered to manifest successful implementation.

## 4. Discussion 

Study circles focusing on eating and nutrition were found to have a positive effect on learning and the workplace context (conceptual use of knowledge). In an earlier study, the study circle intervention was shown to also have positive effects on nutritional care and on care recipients' nutritional status (instrumental knowledge use) [2, 3]. The study circle intervention seems to give quality improvements leading to professional development (learning), better system performance (care), and better patient outcomes (health), by integrating evidence, context, and facilitation in the efforts to achieve KT. 

Different methods were used to evaluate the effects of study circles. In some cases, the findings from using one method were confirmed by using another method. For instance, through the focus group interview some results found through the quantitative continuous evaluations were confirmed. This can be considered a methodological strength. It could be seen as a shortcoming that the results are based on the staff's self-reflections, for instance, when it comes to reflecting on one's own knowledge development retrospectively. Anyhow, this result was indirectly confirmed when comparing the results of those who had participated in the intervention and those who had not, and it was confirmed by the focus group interviews. Another example of what could be studied more directly is the improvements related to the working place context, for instance better working routines and work atmosphere. However, staff members are a part of and create the working place context, and their views are important to capture. In addition, some actual improvements in the provided care were described in the previous studies [2, 3]. Even so, it seems worthwhile to conduct more studies directly focusing on the actual interaction between team members and between staff and care recipients during an intervention with study circles.

In the efforts to achieve a successful implementation, it is of utmost importance to consider the context and the power of teamwork. This was evident in several ways in the results, not the least in the category “socio-culturally mediated knowledge spread.” Thus, the systematic implementation of study circles creates a favourable context, including workplace climate, that also leads to a more positive view among management and staff of what the circles can achieve for individuals, for the team, and for the care recipients. 

Improving teamwork is not easily achieved by traditional methods, and single interventions have limited impact in KT [38, 39]. In this study, using multifaceted interventions, it was found that skills were developed within the study circles, by the teams, and that this in time could develop the overall capacity of the organisation, allowing further improvements. In a study by Zeitz et al. [40], it was found that the complexity behind the so-called “simple care” (providing warm drinks, appetizing food, energy enriched food, and oral supplements) relied on the active management and broader transformation of the system. In order to achieve improvements in such pragmatic issues as giving care recipients good nutrition, organisational aspects had to be tackled, such as teamwork, communication processes, and organisational and individual values and beliefs [40]. When using AOSC as an intervention method, it is important to have homogenous groups and to understand that more study circles in the same workplace can possibly lead to better transformation of the system. AOSC seems to be an optimal pedagogical method that gives priority to the context in order to achieve KT. These conceptual outcomes of KT are most likely generalizable also to other care settings implementing AOSC with focus on nutrition and eating.

The focus on eating and nutrition, and the fact that the staff came together in the study circles, contributed to competence development and led to positive outcomes. Overall, participants experienced a high degree of satisfaction through participating in the study circles. This could be attributed to the format of the study circle, which provided the opportunity for participants to engage in sustained and meaningful discussions about eating and nutrition. It might, however, come as a surprise that the work atmosphere improved through an intervention focusing on improving nutritional care. There could be at least two reasons for this: first of all that the staff came together from the same workplace, and secondly that the focus was on eating and nutrition. In another study, “food” seemed to be a medium through which other themes were discussed, for example respect, teamwork, communication and redesigning systems of care delivery [40]. Thus, the successful conceptual outcome could be due to both the AOSC and the actual focus within these, that is, an integration of context and evidence. This might in turn strengthen the fact that the directed content analysis connected both the PARiHS concept context and evidence to the category “socio-culturally mediated knowledge spread”. Also, Gougoulakis [15] states in a thesis that learning in study circles is socio-culturally conditioned—knowledge is created and grows in the meeting and communication with others in study circles. In this perspective, knowledge is not something that is only registered. Instead, it is developed in collaboration, relates to the participants' context, and a propitious learning atmosphere that comes about when participants have an exchange without competition [15]. 

By attending study circles, individuals felt that they developed their competence. This became apparent in several ways. For instance, when staff retrospectively self-rated their knowledge, before and after attending the study circles, a significant improvement was indicated. In addition, the participants in the SC considered their knowledge more sufficient than what non-participants rated. Thus, in study circles a socio-culturally mediated knowledge spread, including collaborative learning, takes place that can form the basis for KT and quite likely a successful implementation of evidence.

In order to be successful in the KT, staff needed support from project leaders, management, and from trained circle leaders. The intervention needed to be anchored in the management and the management received feedback that in turn made them more eager to support the intervention. Anchoring and feedback (every six months) was provided by the project leaders. Both quantitative and qualitative findings support the need for facilitation. Participants experienced that the management was engaged in and took care of ideas regarding food and mealtimes to a greater extent than what the non-participants experienced. In addition, the need for facilitation was evident in the category “facilitation of knowledge spread and sustainability.” In addition, the staff needed facilitation from the circle leaders. A full-day training in the study circle leader role was effective in helping them to get the group to focus on the tasks for the circle, which in turn most likely enhanced learning in the group. 

There were no experts involved in the study circles, which might have contributed to the high degree of satisfaction, as the power was evenly distributed between participants and symmetric relationships emerged, corresponding to Piaget's ideas about “cooperative relations” [17]. Participants also found it important that kitchen staff attended the study circles. This contributed to better communication between staff at the unit and staff working in the kitchen. From a project leader perspective, there needed to be a balance between structuring the process of study circles and enabling teams to maintain ownership and control. It might be that if the study circles had a more explicit focus on nutritional screening and management of residents with nutritional risk, the instrumental outcomes would have been even better than found in previous studies [2, 3]. However, this could be at the cost of conceptual outcomes, that is, understanding, knowledge, and attitudes. Correspondingly, not only improvements in knowledge but also in the perception of how work was carried out, in the view of care recipients, working atmosphere and cooperation were described. 

The study circle participants and leaders wanted to continue the work, and felt that the work had only just begun—similarly to what was found in a study by Wiechula et al. [39]. Thus, facilitation was emphasised to continue also after the intervention period. also it most likely did continue to some extent, as some positive instrumental outcomes (provision of energy enriched food and/or oral supplements) were seen also one year after the project ended [3]. Thus, facilitation from different levels in the organisation, together with regular evaluations and feedback from project leaders, is likely to contribute to a “learning organisation”, and it needs to continue also after the project has been completed. In the concept “a learning organisation” the two PARiHS concepts facilitation and evidence get connected, as it is also evident in the category “facilitation of knowledge spread and sustainability” found in this study.

By making the study circles action oriented, several positive effects are achieved. Besides the findings in this paper, also a few earlier studies have shown positive effects, both in the care provided and for the care recipients [2, 3, 28]. The PARiHS framework can be used to understand the interactions and complexities involved in KT activity. The positive changes achieved can possibly be attributed to the fact that complex interventions were developed in the study circles that fit the local contexts. Melding and implementing evidence involves negotiation and shared understanding [12]. In addition, the need to consider the context in order to achieve quality improvements as emphasised by Kitson and colleagues [12] is supported by the findings from this study. 

## 5. Conclusions

KT in nursing homes is a complex and challenging activity. Through action-oriented study circles, priority is given to the staff's experiences, feelings, values and beliefs, and teamwork when striving towards quality improvements. Knowledge to action is achieved through socio-culturally mediated knowledge spread, including collaborative learning, and through facilitation of knowledge spread and sustainability in a learning organisation. 

The study circle seems to be an outstanding pedagogical method for getting staff in care and service from the same unit, in the same context, to focus on a specific knowledge area. By making study circles action oriented, several positive effects can be achieved for the staff's learning, team collaboration, care provision, and most importantly, for the care recipients' health. The findings from this study have implications for other care settings implementing AOSC with focus on eating and nutrition.

## Figures and Tables

**Table 1:** Number of study circles and participants every six months.

Period	Number of study circles	Number of participants
Spring 2006	6	49
Autumn 2006	11	92
Spring 2007	14	111
Autumn 2007	7	63
Spring 2008	12	109
Autumn 2008	21	168

Total	71	592

**Table 2:** Characteristics of participants and their evaluation of the study circles.

	*n* = 592
Age, mean (SD)	45.2 (10.0)
Gender, men/women, %	4/96

Profession, %	
Auxiliary nurses or nursing assistants	80
Cook, kitchen helpers	12
Registered nurses	3
Team head, students, dietician	3
Home care, auxiliary nurses	2

Number of years in current workplace, %	
Less than one year	2
One to five years	14
Six to ten years	18
Eleven to fifteen years	6
Sixteen to twenty years	16
More than twenty years	44

Content–interesting, %	
Very interesting	42
Interesting	56
Neither nor	2
Fairly/totally uninteresting	0

Content–relevant, %	
Very relevant	33
Relevant	61
Somewhat relevant	6
Not very/not at all relevant	0

Level of difficulty, %	
Very difficult	0
Difficult	1
Neither difficult nor easy	46
Easy	46
Very easy	7

**Table 3:** Study circle participants' (*n* = 592) retrospective self-evaluation of their knowledge development.

	Study circles		
	Before, %	After, %	*P*-value
Knowledge, %			
I have sufficient/great knowledge	72	96	<0.0005^(1)^
I feel insecure whether my knowledge is sufficient or not	25	3	
I do not have sufficient knowledge	3	1	

^(1)^Wilcoxon signed ranks test.

**Table 4:** Knowledge-to-action goals set within study circles focusing on eating and nutrition.

Goal category	Example of focus
	Create a homelike environment
Mealtime environment	Bake at the unit, fragrance impressions from food, tease the appetite
	Create a calm atmosphere in the dining room

Food	Offer alternatives to oral supplements Spices on the table Individually adapted food and consistencies

Hygiene	Be careful with hand hygiene Keep cold food cold and warm food warm (check temperature) Improve mouth care

Routines	Ask newly admitted about food habits Control weight for those moving in and continually Inform substitute staff about goals and routines

Shorten the overnight fast	Offer evening coffee/tea/sandwich Postpone evening meal Have night staff offer sandwich and milk

	Have the residents serve themselves food
The ability to choose	Have the residents choose between different dishes
	Offer the residents assistance to go to the dining room

Cooperation	Improve cooperation between kitchen, registered nurses and other professions

**Table 5:** Comparisons between staff members who had participated in study circles (*n* = 74) and those not (*n* = 115).

	Study circles		
	Non-participants, *n* = 115	Participants, *n* = 74	*P*-value
Age, mean (SD)	45.7 (9.7)	47.5 (10.0)	0.241^(1)^
Gender, men/women %	3/97	6/94	0.286^(2)^

Profession, %			**<**0.0005^(2)^
Auxiliary nurses or nursing assistants	75	86	
Cook, kitchen helpers	0	11	
Registered nurses	20	3	
Home care, auxiliary nurses	5	0	

Number of years in current workplace, %			0.677^(3)^
Less than one year	3	0	
One to five years	13	8	
Six to ten years	15	16	
Eleven to fifteen years	12	16	
Sixteen to twenty years	10	11	
More than twenty years	47	49	

Knowledge, %			0.044^(3)^
I have sufficient/great knowledge	63	69	
I feel insecure whether my knowledge is sufficient or not	33	31	
I do not have sufficient knowledge	4	0	

The management is engaged in and takes into account ideas regarding food and mealtimes, %			0.009^(3)^
Completely/partly agree	66	80	
Can't decide	23	10	
Completely/partly disagree	11	10	

^(1)^ *t*-test.

^(2)^Chi-square test.

^(3)^Mann Whitney *U* test.

**Table 6:** Inductive results structured into core category, categories, subcategories, and quotations (different quotations are separated by a slash). The PARiHS (Promoting Action on Research Implementation in Health Sciences) framework deduced to the inductive results.

Core category	Categories	Subcategories	Quotations	PARiHS framework
		Together and from the same place we are stronger	After training, when you come back, usually alone, it is hard to get people to listen. It is difficult to adapt the new knowledge to your own workplace. / …initially, we had one participant from each unit, then with participants from the same unit, there was an enormous difference. / If you carry out one study circle at a unit, then you might achieve some things, but it is when the second study circle is formed that you can really achieve changes.	Context
	Socioculturally mediated knowledge spread	Getting together broadens one's perspective and improves the work atmosphere	People have a greater understanding for one another, are more open, and feel free to ask questions about different things. One broadens one's views, one can actually do things differently, if you don't try–nothing happens.	Context
Nutritional knowledge translation expressed as “going from feeding to serving”^(a)^		Through discussions, the gap between knowledge and practice can be bridged	You get a new perspective, think differently than before./ I would absolutely not want to have missed this time, never in my life, I have learned so much that I can make use of. / We will try to figure out everything that we can do, instead of focusing on things we can't do. / A culture can develop that is not justified.	Evidence
		Action-oriented goals	The study circles have been goal-directed, it is easier to explain to the others together what to do. / These documents stating goals…they have led to so many changes at the units. / There is cooperation between all of us: team manager, staff, kitchen, and the actual health care. Everyone works towards the same goal: we have a human being who we want to feel well, and it is our job to do what we can to make it possible.	Evidence
	Facilitation of knowledge spread and sustainability	Facilitation by anchoring and feedback	There has been a lot of persuasion, and a lot of marketing…geared towards team managers and the management group. Then there are the politicians as well.	Facilitation
		Facilitation of sustainability	We have put in a lot of work on this and we have to make sure that it continues even though the study circles end. / It is not supposed to end just because the three years have passed, it is a process that is supposed to continue. / Yes, it is easy to fall back into old habits…there should perhaps be some sort of follow-up. / Finally, we have made a dent at the shortcomings we have had, and now we will continue forward.	Facilitation

^(a)^“Successful implementation” is deduced using the PARiHS framework. The idea for labelling this core category stemmed from an actual statement in the focus group interview, “we are thinking more in terms of service, it is not a matter of feeding anymore.”
